# Exposure to arsenic and level of Vitamin D influence the number of Th17 cells and production of IL-17A in human peripheral blood mononuclear cells in adults

**DOI:** 10.1371/journal.pone.0266168

**Published:** 2022-04-11

**Authors:** Faruque Parvez, Fredine T. Lauer, Pam Factor-Litvak, Tariqul Islam, Mahbubul Eunus, M. Abu Horayara, Mizanour Rahman, Golam Sarwar, Habibul Ahsan, Joseph H. Graziano, Scott W. Burchiel

**Affiliations:** 1 Department of Environmental Health Sciences, Mailman School of Public Health, Columbia University, New York, New York, United States of America; 2 Department of Pharmaceutical Sciences, University of New Mexico College of Pharmacy, Albuquerque, New Mexico, United States of America; 3 Department of Epidemiology, Mailman School of Public Health, Columbia University, New York, New York, United States of America; 4 University of Chicago and Columbia University Field Research Office, Dhaka, Bangladesh; 5 Department of Health Studies, University of Chicago, Chicago, Illinois, United States of America; University of Rajshahi, BANGLADESH

## Abstract

There is limited evidence on the effects of environmental exposure to arsenic (As) on the immune system in adults. In a population-based study, we have found that urinary As (UAs), and its metabolites [inorganic As (InAs), monomethylated arsenicals (MMA^+3/+5^), and dimethylated arsenicals (DMA^+3/+5^)] modulate or influence the number of T-helper 17 (Th17) cells and IL-17A cytokine production. In non-smoking women, we observed that UAs and DMA^+3/+5^ were associated with changes in Th17 cell numbers in a nonlinear fashion. In smoking males, we found that UAs was associated with a significant decrease of Th17 cell numbers. Similar association was observed among non-smoking males. Likewise, UAs, DMA^+3/+5^ and MMA^+3/+5^ were associated with diminished production of IL-17A among non-smoking males. When stratified by Vitamin D levels defined as sufficient (≥20 ng/ml) and insufficient (<20 ng/ml), we found a substancial decrease in Th17 cell numbers among those with insufficient levels. Individuals with sufficient VitD levels demonstrated significant inhibition of IL-17A production in non-smoking males. Collectively, we find that exposure to As via drinking water is associated with alterations in Th17 numbers and IL-17A production, and that these associations may be modified by Vitamin D status. Our findings have significance for health outcomes associated with As exposure.

## Introduction

Environmental exposure to As is a major global health problem as As is associated with cancer [1], lung diseases [2], cardiovascular diseases, childhood neurodevelopment [3, 4], and diabetes [5]. Some beneficial effects of As have also been reported including treating acute promyelocytic leukemia (APL), a type of acute myeloid leukemia (AML) [6–8]. However, human population studies have shown evidence of As toxicity in peripheral blood leukocytes obtained from adults and children exposed to As [9, 10], as well as from cord blood [11, 12].

Few studies address immune function using cell-based assays. Our labs find that As modulates cell function and lymphocyte activation by examining *ex vivo* activated cryopreserved PBMC T-cells [13, 14]. We previously found that As and environmental polycyclic aromatic hydrocarbons (PAHs) modulate lymphocyte subsets and cytokines in male smokers and non-smokers [14]. Recently we found that VitD plays a significant role in modulating the activation and proliferation of T-cells in As–exposed PBMC obtained from non-smoking and smoking men [15].

The purpose of this paper is to examine a specific subset of human T-helper (Th) cells for potential toxicity produced by As exposure *in vivo*. Our previous studies show that Th cell subsets are susceptible to As exposure [13]. Further, *in vitro* exposure of human T-cells to As results in a decrease in IL-17A production [16]. Thus, it was of interest to determine if As-altered IL-17A production and/or Th17 cell numbers could be identified following environmental exposures to As in human populations.

We and others find a high incidence of VitD deficiency in Bangladesh [17]. Therefore, a second goal of these studies was to follow-up on our observations that VitD may modulate As immunotoxicity [15]. VitD deficiencies are associated with cancer and immunosuppression in human populations [18]. Recently, there is interest in the role of VitD in autoimmune diseases [19]. VitD insufficiency results in a failure in sending tolerogenic signals to T-cells and may lead to inappropriate self-directed activation of immunity [20, 21]. At pharmacologic levels of VitD, inhibition of T-cell activation via tolerogenic signaling is known resulting in VitD being suggested as an immunotherapy for rheumatic diseases [22]. VitD level is also a potential explanation for the sex bias for autoimmune diseases in women, as their VitD levels are lower than men [23]. In the present studies, we examined the relationship between Th17 and IL-17A and chronic As exposures, in adults and in those with insufficient and sufficient circulating blood levels of VitD.

## Methods

### Study population

The study protocol was issued ethical clearance by the Bangladesh Medical Research Council (BMRC) and approved by Columbia University’s Institutional Review Board (IRB). Consent forms and recruitment materials were translated into Bengali and back translated into English. A village health worker from the area was made available for any person unable to read the informed consent or requiring explanation of procedures. Each participant provided either verbal or written consent in the presence of a witness. The protocol for analyses of the biological samples was also approved by the Health Science Center’s Human Research Review Committee (HRC HRRC) of the University of New Mexico.

A list of 2,197 potential participants was generated from the Health Effects of As Longitudinal Study (HEALS) central database based on the following eligibility criteria [15]. Eligible participants were healthy men and women between the ages of 35 and 65 years, regardless of smoking status, and living in the study area of Araihazar, Bangladesh. Initial steps in the recruitment process included a home visit by a field team. Upon the visit to the homes of potential participants 492 were deemed ineligible due to death, migration out of the study area, suffering from a serious or from multiple chronic illnesses, illness or symptoms related to immune function disruption, taking medication(s) that might influence immune function, or were not at home. Our field team also found that 803 individuals were using a different source of drinking water (tube well) from what they had reported at the time of initial recruitment to the HEALS. Of the 902 eligible participants, 791 visited the study clinic; other eligible participants (19) missed their appointment. Blood and urine samples were collected from each participant. We performed hematology tests to further exclude those with abnormalities; 25 samples were excluded due to: abnormal blood sugar levels, suffering from urinary tract infection, or lymphocytosis. Additionally, three samples were excluded at time of PBMC isolation due to hemolysis and another three for low cell count and low viability. Thus, a total of 766 PBMC samples were shipped to University of New Mexico using dry nitrogen shippers. Upon thawing of PBMC for T-cell subset and cytokine production assays, 147 samples were found to have viabilities less than 80%. For T-cell subset analysis we were unable initiate cultures or analyze cell staining for 59 samples due to too few cells. Following flow cytometry analysis of T-cell subsets, 323 samples were removed due to evidence of non-stimulation. The T-cell subset analysis consisted of 237 samples which were analyzed for intracellular staining of IL-17A (CD3+, CD4+, IFNγ- and IL-17A+) and are reported as percent of the CD4 positive (CD4+) gated cells. Of 619 PBMC thawed samples twelve lacked adequate cell numbers for establishing cytokine assays therefore 607 samples were assayed for IL-17A cytokine production. As exposure data was missing for one sample leaving resulting in 236 samples analyzed for T-cell subsets and 606 samples for IL-17A cytokines.

### Measurement of arsenic exposure

Urine samples (15 ml) were collected and frozen (-70°C) at the study clinic in Araihazar, Bangladesh and were sent to the Columbia University for assessment of As and As metabolite content. Graphite furnace atomic-absorption spectrophotometry (GFAAS) as previously described [13, 14] was used for the assessment. A colorimetric method based on the Jaffe reaction, was used to quantify urinary creatinine which was to correct UAs and metabolites. All the exposure measures, including total UAs and metabolites were expressed as μg/g of creatinine.

### Collection and cryopreservation of peripheral blood

Approximately 10 ml of blood was collected at the field clinic by technicians proficient in blood collection. Detailed procedures [24] were followed for PBMC isolation, freezing, and storage. Dry shippers (Cryoport, Irvine, CA) that maintain temperature at or below -150°C were used to ship samples from Bangladesh to the United States. Upon arrival to our lab samples were stored in liquid nitrogen until thawed for testing. All samples were analyzed at the end of the study and researchers were blinded to the exposure. As described previously [15], samples were thawed quickly in a 37°C water bath, washed with warmed media, resuspended in 3 ml fresh media and counted. Cell counts and viabilities were acquired with a Nexcelom Cellometer Auto 2000 Cell Viability Counter using acridine orange and propidium iodide (AO/PI; Nexcelom Bioscience, CS2-0106) according to manufacturer’s directions. Cells with viabilities exceeding 80% were used for testing.

### Intracellular marker staining and detection of TH17 T-cell subset

T-cell subsets were stained as previously described [13, 25] with fluorescence labeled antibodies to intracellular cytokines IFNγ, IL-17A, IL-4 and transcription factor FoxP3 in combination with cell surface markers following an overnight rest and subsequent 24hr activation of the T-cell receptor, non-stimulated cells were run in parallel. Briefly, each sample was plated into individual Petri dishes (non-growth coated) at 4x 10^6^ cells in 3.5 ml complete media [RPMI-HEPES Modified (Sigma R5886) + 10% heat inactivated FBS + 2 mM L-glutamine (Gibco, 25030–081) + 100 U /ml penicillin and 100 μg/ml streptomycin (Pen/Strep; Gibco, 15140–122)] and incubated overnight in a humidified, 5% CO_2_, 37°C incubator. Following incubation, each sample was collected into a centrifuge tube, centrifuged, aspirated and resuspended in 720 μl complete media to yield approximately 5.6x10 ^6^cell/ml. Samples were then plated at 1x10^6^ cells/well into flat bottom cell culture plates that were previously coated with 0.5μg/ml (in DPBS^-^) anti- CD3 antibody (eBiosciences; Functional Grade Purified, clone OKT3) or DPBS^-^ for non-stimulated samples. Anti- CD28 antibody (eBiosciences; Functional Grade Purified clone CD28.2) was added to each well of the stimulated samples at a final concentration of 2 μg/ml (in DPBS^-^) and plates were returned to the incubator for 24hr. Following incubation, cytokine transport from the cell was inhibited by adding Brefeldin A to each well at a final concentration of 10 μg/ml and returned to the incubator for an additional 4hr. Samples were collected into cluster tubes containing cold staining buffer [DPBS w/o Ca^2+^ or Mg^2+^ +0.2% heat inactivated FBS + 0.09% sodium azide]. Samples were centrifuged for 10min at 4°C at approximately 240x g, buffer was poured off and cells were brought up in 50 μl Brilliant Stain Buffer (BD Biosciences). A cocktail of cell surface markers (CD3, CD4, CD25, CD69, CD45RO, CD127 and a fixable viability stain) was added to each tube. Samples were incubated for 20 min on ice, washed with cold stain buffer, then fixed and permeabilized (BD Pharmingen Transcription Factor Buffer set (BD biosciences Cat. No. 562574) for 45 min on ice. Samples were washed twice with the wash buffer provided and resuspended in Brilliant Stain Buffer (BD Biosciences). A cocktail of antibodies for intracellular staining of IFNγ, IL-17A, IL-4 and FoxP3 was added to each of the samples (BD Biosciences) and samples were incubated on ice for 45min, then washed twice with the provided wash buffer and resuspended in 400 μl stain buffer. Samples were covered to protect from light, held at 4°C overnight and analyzed the next day on the LSR Fortessa flow cytometer and collected as flow cytometry standard (FCS) data files. FlowJo software v10.6.2 was used to analyze FCS data. T-cell subsets were discriminated using a gating strategy for sequential identification of the following: all cells, single cells, live cells, CD3+, CD4+ and IL-17+ (IFNγ-) cells. Cells were also evaluated for activation by examining the CD69+ as well as the CD25+ cell populations. Upon analysis of CD69+, used to indicate activation, 323 samples were removed from the Th17 study analysis based on indication by CD69 that the samples were not activated.

### IL-17A cytokine assay

As described in our previous work [14], cells were plated at 1x10^5^ cells/well and stimulated as outlined above using anti-CD3 immobilized on a 96 well cell culture plate in combination with addition of solubilized anti-CD28 to the plated cells. Non-stimulated samples were run in parallel DPBS^-^ replaced the anti-CD3 coating and media was substituted for anti-CD28. Plates were incubated for 72hr after which, supernatants were collected from the wells and aliquoted and stored at -80°C until they were assayed using Meso Scale Discovery (MSD) multiplex electrochemiluminescence immunoassay for analysis. IL-17A was detected using a V-Plex, single spot (Human) kit by MSD. Supernatants were thawed, centrifuged at 2000x*g* for 3min at 4°C and then diluted 100-fold (two serial 10-fold dilutions) using complete media. Calibrator dilutions were prepared with 4-fold dilutions following the kit instructions to create an 8-point standard curve. For analytical purposes, samples were assigned a value of one-half the lower detection limit for the assay if they fell below the fit curve or detection range of the instrument.

### Measurement of 25-Hydroxyvitamine D (Vitamin D)

Sample serums were sent to the Department of Medicine in Columbia University’s Medical Center for determination of VitD levels. 25-hydroxyvitamine D, the sum of 25-hydroxyvitamine D2 (25(OH)D_2_) and 25-hydroxyvitamine D (25(OH)D_3_), were assayed by Ultra-Performance Liquid Chromatography- tandem mass spectrometry (LC-MS/MS) as described previously [15]. The level of sensitivity for LC-MS/MS assay was 1.0 ng/ml for both 25(OH)D_2_ and 25(OH)D_3_. Intra-day precision was 2.4% for 25(OH)D_2_ and 3.5% for 25(OH)D_3_. Inter-day precision was 8.1% for 25(OH)D_2_ and 5.5% for 25(OH)D_3_. From the sample serums 25(OH)D_2_ and 25(OH)D_3_ were extracted using liquid-liquid extraction. Chromatographic separation was performed on an Agilent Poroshell 120 EC-C18 column (3.0 x 50mm, 2.7 μm) using a gradient of 70%-90% methanol containing 0.1% formic acid. Multiple reaction monitoring (MRM) of the analyses was performed with positive electrospray ionization with the following MRM transitions: 413->395 for 25(OH)D_2_, 401->383 for 25(OH)D_3_ and 407->389 for d6-25(OH)D_3_. Calibrators were standardized against the NIST standards and the assay passed the proficiency testing of international DEQAS. In the U.S. the normal reference range for vitamin 25(OH)D_3_ is 30–100 ng/mL [26].

### Statistical analysis

As only 7 smoking women were enrolled, we eliminated them from the analysis. Thus, the final dataset consisted of a total of 236 samples for T-cell subset (Th17) analysis and 606 samples for cytokine (IL-17A) analysis. We first calculated descriptive statistics for female and male non-smokers, and male smokers. Group differences were assessed using the Kruskal-Wallis test, with adjustment for pairwise multiple comparisons using Dunn’s Method, for serum VitD, total UAs and UAs species (InAs, MMA^+3/+5^, and DMA^+3/+5^), with all urinary measures adjusted for urinary creatinine. Spearman correlation coefficients describe bivariate associations among quantitative variables. To reduce the impact of extreme variables and improve model fitting, we transformed the exposure variables with right skewed distributions using logarithmic function.

Linear regression models estimated the associations between each exposure variable (i.e. total urinary As and the urinary As metabolites) and the outcomes of Th17-cells and IL-17A cytokine production, adjusting for age and body mass index (BMI). These models were developed in several steps. First, individual models were fit for female non-smokers, male non-smokers and male smokers, all adjusting for age and BMI. In a second step, we added circulating serum VitD into the linear regression models. Third, to test for effect modification between VitD status and UAs/UAs species, we divided Vit D concentrations into low/insufficient serum levels (<20 ng/ml) and high/sufficient levels (>20 ng/ml). Separate models relating UAs/UAs species to Th17 or IL 17A were fit for each Vit D stratum.

Finally, generalized additive models (GAM) were used to evaluate possible non-monotonic relationships between UAs/UAs species and Th17 cell subset or IL-17A production. GAMs allow for both a parametric component of exposure (which assesses a linear relationship) and a non-parametric component, which assesses non-monotonicity. Based on the results of the GAM models, we fit linear models for the immune parameter outcomes with the most parsimonious polynomials of the exposure variables to best describe the patterns of the associations. SAS version 9.4 was used for statistical analysis.

## Results

### Characteristics of the study cohort

The study cohort was comprised of 614 men and women [15]. Peripheral blood mononuclear cell (PBMC) samples from 236 participants were analyzed for the Th17 T-cell subset (Table 1), and 606 PBMC samples from individuals were included for IL-17A cytokine analysis (Table 2). The study cohort for Th17 cells and IL-17A cytokine production analyses were comprised of approximately half (52%) non-smoking women. Non-smoking men (12%) and smoking men (36%) made up the other half. Individuals were age 47–53 years with women younger (48 yr) compared to males (52 yr). Women also had higher average BMIs (24) compared to men (22). All participants had similar average UAs concentrations (124–144 μg/g creatinine) except non-smoking men who tended to have lower, although not significantly different, concentrations (95–105 μg/g creatinine). Non-smoking men also had lower average concentrations of InAs (11 μg/g creatinine) as well as the MMA^+3/+5^ (12 μg/g creatinine) metabolite. All participants had similar average of Th17-cell numbers, (reported as %CD4+ cells) and IL-17A production. Men had significantly higher serum VitD levels (26 ng/ml) compared to women (20 ng/ml). Overall, demographics of those included in this study of Th17-cell subset and cytokine production compared to the demographics of the study cohort [15].

**Table 1:** Demographic of the study participants, urinary arsenic species and Th17 cells.

	All samples	Non-smoking Women	Non-smoking Men	Smoking Men
Th17	Mean (SD)	Mean (SD)	Mean (SD)	Mean (SD)
	Median (Range)	Median (Range)	Median (Range)	Median (Range)
**Demographic**	N = 236	N = 122	N = 29	N = 85
Age (years)	50.1 (7.0)	47.6 (6.6)	51.8 (6.6)**	53.0 (6.5)***
	50(38–65)	46 (38–64)	51 (40–63)	54 (39–65)
Sex (%)				
Women	52			
BMI	23.0 (4.2)	24.2 (4.3)	22.1 (3.1)*	21.7 (3.9)***
	22.8 (14.3–39.7)	24.1 (14.3–39.7)	21.8 (17.5–29.7)	21.1 (16.0–33.1)
% of cohort		52	12	36
**Average Arsenic Exposure Conc. (μg/g)**				
Urinary As	131.2 (137.9)	144.4 (148.7)	94.7 (81.0)	124.8 (135.6)
	85 (15–827.2)	93.4 (17.6–783.7)	69.5 (15–357.1)	72.1 (22.1–827.2)
Inorganic As	13.5 (17.7)	13.3 (15.4)	11.1 (15.4)	14.6 (21.2)
	7.5 (0–134.8)	8.4 (0–87.3)	6.7 (1.1–80.5)	6.8 (1.3–134.8)
MMA^+3/+5^	16.7 (23.5)	16.3 (22.5)	12.6 (16.6)	18.9 (26.6)
	8.3 (1.6–191.2)	8.2 (2–149.8)	8.0 (1.6–83.2)	9.9 (2.2–191.2)
DMA^+3/+5^	82.1 (84.1)	93.3 (96.8)	58.9 (45.5)	74.0 (71.7)
	51.6 (109–508.9)	58.6 (16.6–508.9)	45.5 (10.9–205.8)	49.2 (14.3–414.0)
Th17 cells (%CD4+ cells)	0.631 (0.70)	0.681 (0.84)	0.568 (0.32)	0.583 (0.55)
	0.440 (0–5.590)	0.450 (0.017–5.590)	0.520 (0.110–1.630)	0.400 (0–3.160)
Vitamin D (ng/ml)	23.1 (7.3)	19.9 (5.6)	26.5 (8.8)***	26.4 (7.0)***
	22.0 (7.6–53.8)	19.6 (7.6–44.1)	24.5 (11.6–53.8)	25.8 (11.9–45.5)

Significance level

***p<0.001,

**p = 0.01,

*p<0.05 Non-smoking women compared to Non-smoking men and to Smoking men.

Significance level

^###^p<0.001,

^##^p = 0.02,

^#^p<0.05

Non-smoking men compared to Smoking men.

**Table 2:** Demographic of the study participants, urinary arsenic species and IL-17A cytokine.

	All samples	Non-smoking Women	Non-smoking Men	Smoking Men
IL-17A cytokine	Mean (SD)	Mean (SD)	Mean (SD)	Mean (SD)
	Median (Range)	Median (Range)	Median (Range)	Median (Range)
**Demographic**	N = 606	N = 311	N = 81	N = 214
Age (years)	50.0 (7.4)	47.4 (7.0)	51.8 (6.9)***	53.0 (6.7)***
	50 (35–65)	46.0 (35–64)	52 (37–65)	53.5 (38–65)
Sex(%)				
Women	51			
BMI	23.1 (4.1)	24.0 (4.3)	22.5 (3.7)**	21.9 (3.7)***
	22.7 (14.0–43.3)	23.6 (14.3–43.3)	22.2 (14.0–37.0)	21.2 (15.4–34.5)
% of Study Cohort		51.3	13.4	35.3
**Average Arsenic Exposure Conc. (μg/g)**				
Urinary As	134.5 (141.7)	140.8 (134.4)	105.1 (103.0)**	136.6 (162.3)
	89.3 (15.0–1640.4)	97.2 (17.6–783.8)	77.5 (15.0–668.9)	81.2 (15.6–1640.4)
Inorganic As	13.8 (17.8)	13.2 (15.3)	10.7 (13.5)	15.9 (22.0)^#^
	7.7 (0–195.9)	7.5 (0–105.7)	6.7 (1.1–80.5)	8.2 (1.1–195.9)
MMA^+3/+5^	16.4 (24.0)	15.1 (20.1)	12.1 (14.8)	20.0 (30.6)^##^
	8.3 (1.6–304.2)	7.9 (1.7–149.8)	7.8 (1.6–83.2)	9.2 (1.8–304.2)
DMA^+3/+5^	86.6 (91.2)	91.7 (87.9)	68.3 (60.7)**	86.0 (104.2)
	56.8 (9.5–1136.4)	62.1 (9.6–508.9)	45.5 (9.5–334)	52.8 (12.1–1136.4)
IL-17A (pg/ml)	1987 (1703.7)	2087.4 (1797.8)	2025.9 (1476.6)	1826.9 (1637.7)
	1536 (24–16623)	1598.5 (24–16623)	1679 (149–8206)	1311.5 (70–9576)
Vitamin D (ng/ml)	22.6 (7.4)	19.3 (5.9)	25.6 (7.2)***	26.4 (7.1)***
	21.8 (5.9–53.8)	19 (5.9–44.1)	25.2 (11.2–53.8)	26.0 (11.9–50.6)

Significance level

***p<0.001,

**p = 0.01,

*p<0.05 Non-smoking women compared to Non-smoking men and to Smoking men.

Significance level

^###^p<0.001,

^##^p = 0.02,

^#^p<0.05 Non-smoking men compared to Smoking men.

### Effects of urinary arsenic and urinary arsenic species on the Th17 T-cell subset

Multivariable regression analysis, adjusted for age and BMI, indicated that there were significant inverse associations between UAs and DMA exposures and Th17-cells in all participants and in smoking men (Table 3). When we evaluated the linearity of the relationships, we found evidence of nonlinear relationships for the associations between total UAs and urinary DMA and Th17 in non-smoking women. In this group, the best fitting model included the linear term (on a log scale), and the quadratic and the cubic terms such that there was a strong positive linear association, a negative quadratic association and a small cubic association (Table 3). In non-smoking males, there were inverse associations between UAs and DMA exposure and Th17 cells; however, they were not statistically significant.

**Table 3:** Association between urinary arsenic species and Th-17 cells and IL-17A cytokine production for all participants and by sex and smoking status.

	All participants^a^		Non-smoking Women		Non-smoking Men		Smoking Men	
	B (95% CI)	p-value	B (95% CI)	p-value	B (95% CI)	p-value	B (95% CI)	p-value
**Th17**	**(n = 236)**		**(n = 123)**		**(n = 29)**		**(n = 86)**	
Urinary As^b^^,^^c^	**-0.15 (-0.30, -0.01)**	**0.04**	**14.0 (-0.445, 28.4)**	**0.06**	-0.19 (-0.47, 0.09)	0.20	**-0.22 (-0.46, 0.02)**	**0.08**
Urinary As^2^			**-3.07 (-6.09, -0.0516)**	**0.05**				
Urinary As^3^			**0.217 (.00924, 0.425)**	**0.04**				
Inorganic As ^b^	-0.09 (-0.22, 0.04)	0.18	-0.04 (-0.24, 0.16)	0.69	-0.16 (-0.39, 0.08)	0.20	-0.13 (-0.33, 0.07)	0.21
MMA^+3/+5^ ^b^	-0.09 (-0.22, 0.04)	0.16	-0.07 (-0.27, 0.12)	0.46	-0.15 (-0.39, 0.09)	0.23	-0.09 (-0.30, 0.11)	0.39
DMA^+3/+5^ ^b^^,^^c^	**-0.14 (-0.29, 0.01)**	**0.06**	**18.3 (1.91, 34.7)**	**0.03**	-0.19 (-0.49, 0.11)	0.23	**-0.21 (-0.45, 0.03)**	**0.10**
DMA^+3/+5 (2)^			**-4.24 (-7.92, -0.555)**	**0.03**				
DMA^+3/+5 (3)^			**0.317 (0.0465, 0.587)**	**0.02**				
**IL-17A cytokine**	**(n = 606)**		**(n = 311)**		**(n = 81)**		**(n = 214)**	
Urinary As^b^	**-0.10 (-0.19, -0.01)**	**0.03**	-0.05 (-0.18, 0.07)	0.40	**-0.25 (-0.49, -0.01)**	**0.04**	-0.12 (-0.26, 0.03)	0.13
Inorganic As ^b^	-0.06 (-0.13, 0.02)	0.16	0.03 (-0.08, 0.13)	0.63	-0.16 (-0.36, 0.04)	0.13	-0.10 (-0.23, 0.03)	0.12
MMA^+3/+5^ ^b^	**-0.09 (-0.17, -0.02)**	**0.02**	-0.05 (-0.16, 0.06)	0.37	**-0.20 (-0.41, 0.02)**	**0.07**	-0.10 (-0.23, 0.03)	0.12
DMA^+3/+5^ ^b^	**-0.12 (-0.21, -0.03)**	**0.007**	-0.08 (-0.21, 0.04)	0.21	**-0.28 (-0.51, -0.05)**	**0.02**	-0.13 (-0.28, 0.02)	0.1

^*a*^ Excluding 7 smoking women

^*b*^ Linear regression models were run separately for different arsenic exposure measures (log transformed) and adjusted for Age and BMI

^*c*^ Based on analysis of splines, resulting in polynomial regressions (see text).

### Effects of urinary arsenic and urinary arsenic species on IL-17A cytokine production

Multivariable regression analyses adjusted for age and BMI, showed statistically significant inverse associations between UAs (p = 0.03), MMA^+3/+5^ (p = 0.02) and DMA^+3/+5^ (p = 0.007) (Table 3) exposures and IL-17A cytokine production in all participants, mostly driven by associations in non-smoking men.

### Effects of urinary arsenic on the Th17 cells by VitD status

To further examine the associations between As exposure and Th17 cells by VitD, we stratified VitD by low (<20 ng/ml) and high serum VitD (>20 ng/ml). Table 4 shows, that in both male and females (all participants; n = 236), Th17 was inversely associated with UAs (p<0.05), MMA^+3/+5^ (p = 0.07) and DMA^+3/+5^ (p<0.05) in the low VitD strata.

**Table 4:** Association between urinary arsenic species and Th17 cell subset stratified by low and high Vitamin D levels.

	All participants (n = 236)	Non-smoking Women (n = 123)	Non-smoking Men (n = 29)	Smoking Men (n = 86)
	B (95% CI); p-value	B (95% CI); p-value	B (95% CI); p-value	B (95% CI); p-value
**Exposure**^a^ **and VitD**^b^				
**Urinary As**				
Low VitD	**-0.42 (-0.74, -0.10); 0.01**	-0.39 (-0.80, 0.02); 0.07	-0.31 (-1.33, 0.71); 0.59	-0.97 (-2.03, 0.10); 0.10
High VitD	-0.03 (-0.18, 0.11); 0.64	0.02 (-0.20, 0.24); 0.88	-0.14 (-0.42, 0.14); 0.33	-0.07 (-0.31, 0.16); 0.55
**InAs**				
Low VitD	-0.20 (-0.46, -0.06); 0.14	-0.11 (-0.45, 0.22); 0.50	-0.18 (-1.01, 0.64); 0.69	-0.64 (-1.68, 0.41); 0.26
High VitD	-0.03 (-0.16, 0.10); 0.69	0.02 (-0.20, 0.23); 0.89	-0.11 (-0.34, 0.12); 0.34	-0.02 (-0.22, 0.19); 0.87
**MMA** ^+3/+5^				
Low VitD	**-0.27 (-0.56, 0.02); 0.07**	-0.24 (-0.62, 0.14); 0.22	-0.13 (-0.97, 0.70); 0.78	-0.62 (-1.80, 0.57); 0.33
High VitD	-0.01 (-0.14, 0.11); 0.82	0.001 (-0.19, 0.19); 1.0	-0.17 (-0.40, 0.07); 0.18	0.01 (-0.18, 0.21); 0.90
**DMA** ^+3/+5^				
Low VitD	**-0.37 (-0.69, -0.04); 0.03**	-0.30 (-0.71, 0.11); 0.16	-0.35 (-1.36, 0.67); 0.55	-0.87 (-1.89, 0.16); 0.13
High VitD	-0.03 (-0.18, 0.11); 0.64	0.01 (-0.21, 0.24); 0.90	-0.13 (-0.44, 0.18); 0.42	-0.08 (-0.31, 0.16); 0.53

^*a*^Linear regression models were run separately for each exposure measure (log transformed) adjusted for Age and BMI.

^*b*^VitD level: low/deficient ≤20 ng/ml; high/sufficient >20 ng/ml.

### Effects of urinary arsenic and urinary arsenic on the IL-17A production stratified by VitD

In the high VitD group, we found significant associations between urinary As and all metabolites and IL-17A cytokine inhibition (Table 5). These associations were stronger and statistically significant in non-smoking men for UAs and DMA^+3/+5^. In general, point estimates were greater for men (regardless of smoking status) than for non-smoking women.

**Table 5:** Association between urinary arsenic species and IL-17A cytokine production stratified by low and high Vitamin D levels.

	All participants (n = 603)	Non-smoking Women (n = 310)	Non-smoking Men (n = 80)	Smoking Men (n = 213)
	B (95% CI); p-value	B (95% CI); p-value	B (95% CI); p-value	B (95% CI); p-value
**Exposure**^a^ **and VitD**^b^				
**Urinary As**				
Low VitD	-0.03 (-0.16, 0.10); 0.63	-0.04 (-0.20. 0.12); 0.60	-0.18 (-0.59, 0.23); 0.40	-0.01 (-0.30, 0.28); 0.95
High VitD	**-0.13 (-0.25, -0.01); 0.03**	-0.06 (-0.25, 0.14); 0.58	**-0.28 (-0.56, -0.01); 0.05**	-0.15 (-0.33, 0.02); 0.09
**InAs**				
Low VitD	0.03 (-0.08, 0.13); 0.64	0.09 (-0.04, 0.22); 0.18	-0.14 (-0.48, 0.19); 0.41	-0.07 (-0.32, 0.18); 0.60
High VitD	**-0.11 (-0.21, -0.001); 0.05**	-0.05 (-0.23, 0.13); 0.57	-0.16 (-0.40, 0.08); 0.20	-0.11 (-0.27, 0.05); 0.17
**MMA** ^+3/+5^				
Low VitD	-0.04 (-0.15, 0.07); 0.45	-0.02 (-1.36, 1.33); 0.78	-0.15 (-0.51, 0.21); 0.43	-0.06 (-0.30, 0.18); 0.60
High VitD	**-0.12 (-0.22, -0.01); 0.03**	-0.07 (-0.24, 0.11); 0.46	-0.21 (-0.46, 0.03); 0.09	-0.11 (-0.26, 0.04); 0.15
**DMA** ^+3/+5^				
Low VitD	-0.03 (-0.16, 0.10); 0.63	-0.05 (-0.21, 0.11); 0.53	-0.12 (-0.53, 0.29); 0.57	0.005 (-0.31, 0.32); 0.98
High VitD	**-0.16 (-0.28, 0.05); 0.007**	-0.10 (-0.30, 0.10); 0.34	**-0.36 (-0.62, -0.10); 0.01**	-0.16 (-0.34, 0.01); 0.07

^*a*^Linear regression models were run separately for each exposure measure (log transformed) adjusted for Age and BMI.

^*b*^VitD level: low/deficient ≤20 ng/ml; high/sufficient >20 ng/ml.

## Discussion

Arsenic is a naturally occurring global environmental metal toxicant that is associated with significant morbidity and mortality [27]. The immune system is an important target of As and numerous diseases may be associated with altered innate and adaptive immunity. Host resistance to infection and anti-tumor immunity is suppressed by exposure of human populations to As. Immunosuppression may also be associated with cancers induced by As [28]. Arsenic exists in both a pentavalent (+5) and trivalent (+3) inorganic state that is metabolized by arsenite-3-methyl transferase (As3MT) [29]. Metabolism of As to monomethylated arsenicals (MMA^+3/+5^) and dimethylated arsenicals (DMA^+3/+5^) play important roles of increasing clearance of As [29]. However, the trivalent forms of MMA and DMA may also have more toxicity than InAs [30, 31]. Arsenic metabolism varies from person to person [32] and is linked to differences in susceptibility to diseases induced by As [33]. Additionally, there appear to be important sex differences in the action of As in males and females that may differ by disease and organ system. Based on skin lesions/cancer [34, 35] and pulmonary disease [36], males appear to be more sensitive to the toxicity of As than women. The reason for this increased sensitivity is not clear, but may in part relate to metabolic, nutritional status and exposure differences between the sexes.

The present studies were conducted to evaluate the effects of As on a population exposed chronically to a large concentration range of As in drinking water, some with UAs concentrations in excess of 1,000 μg/g creatinine. We have previously found that As exposures alter lymphoid subsets in the PBMC fraction and that *ex vivo* functional changes can be detected in cell activation markers on B and T-cells, as well as cytokines produced by lymphocytes and monocytes [14]. In these prior studies Th17 cells were increased by As. However, in the present studies we see an inverse association in males who smoke indicating Th17 cells were decreased by As. We also show that the As mediated association is nonlinear in the non-smoking females. The non-monotonic curves associated with As in non-smoking women indicates complex exposure response relationships for Th17cells. Previous studies have shown that IL-17A and Th17 cells are altered in males exposed to As as well as cigarette smoke [13]. However, we have not previously examined female non-smokers and smokers exposed to As. In the present studies, we found that PBMC obtained from males contained fewer Th17 cells and less IL-17A in cell supernatant following activation. However, this was not the case in PBMC from women. Unfortunately, there were inadequate numbers of female smokers to directly compare male and female smokers as well as smoking and nonsmoking females. In both males and female non-smokers, we found that total UAs and DMA^+3/+5^ correlated with a decrease in Th17 cell amounts and IL-17A production. Thus, it appears that exposure to both inorganic and organic forms of As *in vivo* are toxic to Th17 in PBMC. This is perhaps not surprising since direct addition of As to PBMC *in vitro* was found to suppress IL-17A production by activated T-cells [16].

Based on a recent study in which we showed that VitD is an important modifier of As action on the immune system of males [15], we examined potential effect modification by VitD status. We observed a weak correlation between UAs and circulating VitD. Here, we find that Th17 cells were significantly decreased by UAs, MMA^+3/+5^ and DMA^+3/+5^ in study participants who were deficient in VitD. This result is similar to our previous work on T -cell proliferation (TCP) [15, 37]. Conversely, in participants with sufficient levels of VitD we observed UAs, DMA^+3/+5^, and MMA^+3/+5^ exposures were associated with decreased IL-17A production, most notably in the non-smoking males. These results suggest that adequate levels of VitD are needed for arsenicals to interfere with cell signaling pathways required for IL-17A production. VitD is known to modulate T-cell receptor (TCR) activation in human T cells [20]. VitD has also been found to be immunomodulatory in a number of settings [38]. Therefore, As and/or its metabolites may interact with VitD in unexpected ways, such as by modifying T-cell signaling leading to T-cell tolerance or anergy that prevents IL-17A production, as As has been shown to activate certain TCR pathways by increasing Lck and Fyn protein tyrosine kinases (PTKs) required for T-cell signaling [39]. The mechanism responsible for signaling may be due to oxidative stress-related inhibition of protein tyrosine phosphatases (PTPases) that inhibit PTK activity. T-cells given excess signals via PTKs receive unbalanced signals and are not capable of responding to antigen stimulation culminating in cytokine production such as IL-17A. One of the limitations in this study is that we could only examine the role of VitD. It would be interesting to include vitamins such as C and E in future studies, as Vitamin C has be shown to be influential in IL17 production [40].

Human PBMCs treated with environmentally relevant doses of As^+3^ *in vitro* show minimal changes. However, it is the monomethyl metabolite of arsenite (MMA^+3^) that seems to be responsible for suppression of TCP [37]. In human studies, As exposure has been associated with alterations in immune function, including suppression of innate and adaptive immunity [41, 42], perhaps leading to increased cancer [43], and increased upper airway infections [44]. Our studies have shown that As can elevate IL-1β [14], suggesting that As exposure induces a proinflammatory state. Depending upon the dose, duration, and timing of exposure, As may elevate certain immune biomarkers, including some human antibody subsets [45, 46] and cytokines, including Th2-mediators- interleukin (IL)-4, IL-5, IL-13, and eotaxins [47]. Thus, the effects of As on the human immune system *in vivo* is quite complex and its effects can also depend on other environmental and nutritional conditions.

The consequences of As-induced inhibition of Th17 and IL-17A are unclear at this time. IL-17A plays many roles in immunoregulation and has been associated with chronic inflammatory diseases and autoimmunity [48, 49]. Further long-term studies are needed to determine if As is associated with chronic inflammation or autoimmune conditions. It is unclear why males are more susceptible to immune effects associated with the IL-17A axis. The role of methylated As metabolites, and their mechanism of action needs to be further investigated as well.

In summary, our studies show that UAs, MMA^+3/+5^, and DMA^+3/+5^ are associated with decreased numbers of Th17 cells and inhibition of IL-17A production. IL-17A production is more pronounced in PBMC obtained from exposed males than females, and particularly in non-smoking males. The decrease in Th17 cells and suppression of IL-17A are differentially modified by VitD, Th17 cell number is most affected at low or insufficient (<20 ng/ml) levels of VitD, whereas, suppression of IL-17A production is most noted at sufficient (>20 ng/ml) levels of VitD. These findings demonstrate that exposure-response relationships for arsenicals on the immune system may depend on other co-factors and nutritional factors that can exert complex outcomes on adaptive and innate immunity.
